# Prevalence and Extent of Subclinical Atherosclerosis and Associated Cardiovascular Risk Factors in Adult Patients With Psoriatic Arthritis: A Systematic Review

**DOI:** 10.7759/cureus.16853

**Published:** 2021-08-03

**Authors:** Maria Jamil, Reema Aslam, Aanal Patel, Bakhtawar Nadir, Safeera Khan

**Affiliations:** 1 Internal Medicine, California Institute of Behavioral Neurosciences & Psychology, Fairfield, USA; 2 Pediatrics, California Institute of Behavioral Neurosciences & Psychology, Fairfield, USA; 3 Hepatology, California Institute of Behavioral Neurosciences & Psychology, Fairfield, USA; 4 Neurological Surgery, California Institute of Behavioral Neurosciences & Psychology, Fairfield, USA

**Keywords:** psoriasis, psoriatic arthritis, skin inflammation, arthritis, skin diseases, atherosclerotic cardiovascular disease, cardiovascular disease risk factors, atherosclerosis

## Abstract

Psoriatic arthritis (PsA) is a chronic T cell-mediated inflammatory condition affecting a considerable proportion of psoriasis (PSO) patients and a small segment of the general population. Recent studies have shown that patients with PsA are prone to premature atherosclerosis and are at an increased risk of cardiovascular disease (CVD) events, but the extent and prevalence of this are unknown. Our objective was to evaluate the prevalence and extent of subclinical atherosclerosis by measuring the intima-media thickness (IMT) of arteries in adult patients with PsA, as well as identify cardiovascular (CV) risk factors associated with PsA. An extensive literature search was conducted using PubMed as our main database. The articles exploring the association between PsA and subclinical atherosclerosis were included. We also searched other databases like MEDLINE and PubMed Central (PMC). A total of 2,561 studies published between 2005-2021 were obtained by searching the databases, and after the screening process, a total of nine studies were included for review and an additional 22 studies for comparison and backup evidence. As for results, our review included a total of 542 patients with PsA from nine different studies. All the reviewed studies showed a significant association between subclinical atherosclerosis and PsA, as endothelial functions were found to be impaired in PsA patients as deduced by measuring the carotid intima-media thickness (CIMT). PsA patients exhibited greater IMT than healthy controls. Increased IMT independently correlated with parameters of disease activity and conventional risk factors of atherosclerosis. An increased prevalence of CV risk factors such as hypertension, diabetes, obesity, and metabolic syndrome was also found in PsA patients.

## Introduction and background

Psoriatic arthritis (PsA), a common disease with extensive clinical variance, is a chronic inflammatory arthropathy. Approximately 40% of patients with psoriasis (PSO) suffer from PsA [1]. Many chronic inflammatory arthropathies such as rheumatoid arthritis and systemic lupus erythematosus (SLE) have been strongly linked to accelerated atherosclerosis, but there is extraordinarily little data on this association with PsA [2]. Some studies have shown increased mortality rates in PsA patients due to cardiovascular system (CVS) or respiratory causes. Evidence shows a 50% increased risk of cardiovascular (CV) mortality in patients with severe PSO requiring hospitalization. This increased risk could be attributed to several factors, including hypertension, decreased exercise, hyperlipidemia, and chronic inflammation leading to elevated inflammatory levels [3].

However, it is still unclear whether this increase in CVS mortality is due to the CVS risk factors alone. It has been thought that premature occurrence of atherosclerosis related to PSO may be one of the reasons for increased cardiovascular disease (CVD) risk, and some studies have shown an impaired vascular endothelial function in PSO patients. However, very few studies with regard to the extent of subclinical atherosclerosis in PsA patients, in particular, have been carried out [4]. High-resolution carotid ultrasonography is used to obtain measurements of the thickness of the intima and media of carotid arteries; many studies have shown a positive link between increased thickness and atherosclerosis [5]. Medial intima thickness is an independent risk factor for myocardial infarction (MI) and stroke in patients with other, more traditional CVD risk factors [6]. Our Review article focuses on assessing the extent and prevalence of atherosclerosis by measuring the intima-media thickness (IMT) of common carotid arteries in adult patients with PsA.

## Review

Methods

Protocol

We adhered to the Preferred Reporting Items for Systematic Reviews and Meta-analysis (PRISMA) guidelines for carrying out our systematic review [7].

Data Source and Strategy

We searched PubMed, PubMed Central, and MEDLINE (National Library of Medicine) for articles published from 2006 to 2021. We explored the database by using terms of Medical Subject Headings (MeSH) and keywords: "Psoriasis," "psoriatic arthritis," "skin inflammation," "arthritis," "skin diseases," "atherosclerotic cardiovascular disease," "cardiovascular disease risk factors," "atherosclerosis," and "adult," separately and in combination to find relevant studies.

Also, this search was reviewed for relevance. Records were analyzed based on the title and appropriate abstract and were filtered. At the end of our search, we eliminated all duplicate articles. We performed a non-automated search on the reference lists of included studies and systematic reviews.

Study Selection and Eligibility Criteria

Inclusion criteria: there was no language restriction; we included studies in English, Turkish, French, and Spanish. Randomized control trials (RCTs), cross-sectional studies, case-control studies, cohort studies, systematic reviews, traditional reviews, opinions, and animal studies were all included. We included studies published in the last 15 years. We chose only those studies involving humans of age 18 years and onwards in our research.

Exclusion criteria: gray literature, books, documents, overlapping studies, duplicate studies, and studies before 2006 were excluded.

Data Extraction

All titles, abstracts, and full-text articles were screened by two reviewers independently (RA, MJ). The items extracted from each study included the year of publication, sample size, age range, response rate, study design, and study outcome. The studies gathered by one reviewer were also scrutinized by other reviewers for accuracy and eligibility. In case of disagreement, conflicts were resolved by a mutual discussion on the study in question.

The following tools were used to assess the quality of included studies:

• The Newcastle-Ottawa scale - observational/non-randomized controlled trial

• The Scale for the Assessment of Narrative Review Articles (SANRA) checklist - traditional review articles 

• The assessment of multiple systematic reviews (AMSTAR) checklist - systematic review and meta-analysis

After a thorough screening and quality check, nine finalized articles were included for review. Only those articles that satisfied >70% of the checklist quality parameters were included in the review.

Results

A total of 2,561 studies were obtained by searching the databases. After the application of our inclusion and exclusion criteria, we studied a total of 91 articles that were then filtered further. We removed all duplicate studies. After setting a 70% benchmark, we assessed 70 studies for quality, and only nine ultimately qualified to be included in the final analysis after applying the quality assessment tools.

**Figure 1:**
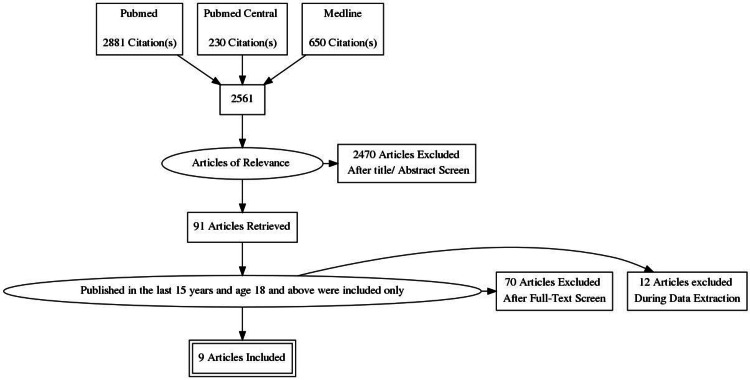
PRISMA flow diagram outlining the search process PRISMA: Preferred Reporting Items for Systematic Reviews and Meta-Analyses

Our study selection strategy is summarized in Figure 1.

We reviewed the nine shortlisted articles, and we used some other published articles (other than the nine selected studies) to support and compare the evidence [1-6,8-32]. The method of retrieving the other studies was by carrying out an extensive search using keywords such as "psoriasis", "subclinical atherosclerosis", "cardiovascular risk factors", etc. Keywords were identified by isolating the subject terms mentioned most frequently. Articles that matched during the extensive search displayed the keywords in relevant areas where further information could be extracted with citation. Our review included 542 patients with PsA from nine different studies [1-4,6,14,17,20,21], and these nine studies comprised four case-control studies, three cross-sectional studies, and two systematic reviews. The main characteristics of the included studies are summarized below in Table 1.

**Table 1:** Summary of nine selected studies CIMT: carotid intima-media thickness; CT: computed tomography; CVS: cardiovascular system; DBP: diastolic blood pressure; DMARDs: disease-modifying antirheumatic drugs; PsA: psoriatic arthritis; PWV: pulse wave velocity; RA: rheumatoid arthritis; SBP: systolic blood pressure

Primary author/year of study	Type of study	No. of patients	Purpose of the study	Intervention studied	Results/conclusions
Şule Apraş Bilgen/2018 [1]	Parallel group study	90	To assess the presence of subclinical atherosclerosis in patients with PsA compared with RA patients and healthy controls	Flow-mediated dilation (FMD) and CIMT	The FMD percentage was significantly smaller in both the PsA and the RA patients compared to healthy controls (p<0.001). The median CIMT was greater in the RA patients compared with the PsA patients and the healthy controls. In conclusion, there was an increased risk of impaired endothelial function in the PsA patients
Oded Kimhi/2007 [2]	Cross-sectional study	147	To evaluate the extent of subclinical atherosclerosis in patients with PsA and to identify vascular risk factors associated with PsA	Carotid duplex ultrasound	The average IMT (mean ± standard deviation) for PsA patients was significantly higher compared with controls (0.76 ± 0.11 versus 0.64 ± 0.27, respectively, p<0.00001). PsA patients exhibited greater IMT than healthy controls. IMT increased independently
Mehmet Soy/2009 [3]	Cross-sectional study	64	To investigate arterial distensibility in patients with psoriasis and PsA by measuring the PWV	Automatic carotid-femoral PWV measurement using the Complior Colson device (Alam Medical, Saint-Quentin-Fallavier, France)	The carotid-femoral PWV is increased in patients with psoriasis and PsA. Mean PWV, SBP, and DBP were significantly higher in psoriatic patients than in control subjects (p=0.036, p<0.001, and p=0.005 respectively)
Yiu KH/2012 [4]	Cross-sectional study	70	The prevalence and extent of atherosclerosis in patients with psoriasis	Coronary calcification score (CCS) measured by multi‐detector CT. Carotid atherosclerosis by high‐resolution ultrasound‐derived CIMT	Patients with psoriasis had a higher prevalence of coronary atherosclerosis (CCS >0; 28.6% vs. 3.9%, p<0.01), and a higher degree of coronary atherosclerosis estimated by the mean CCS (67.4 ± 349.2 vs. 0.5 ± 3.0, p<0.05) compared with controls. Similarly, CIMT was significantly greater in patients with psoriasis than in control subjects (0.73 ± 0.11 mm vs. 0.67 ± 0.08 mm, p<0.01)
Lihi Eder/2008 [6]	Case-control study	80	The prevalence of subclinical atherosclerosis in patients with psoriasis	Duplex scan	The overall prevalence of atherosclerosis was higher among the patients than the controls. Patients with PsA exhibited greater carotid IMT than controls, 1.04 ± 0.35 mm vs. 0.88 ± 0.29 mm, respectively (p=0.03)
Ivan Gruev/2015 [14]	Case-control study	50	To estimate the correlation between subclinical atherosclerosis and traditional CVS risk factors in patients with PsA	Carotid ultrasound (b-mode)	Patients with PsA had higher values of IMT and more plaques on the background of chronic inflammation despite their better CV risk profile compared to hypertensive controls
Serhad Bilim/2021 [17]	Case-control study	101	To evaluate subclinical atherosclerosis using the ankle-brachial index (ABI) in patients with PsA	A Doppler probe and a standard blood pressure cuff were used to calculate the ABI values for each participant	Patients had lower overall ABI (1.03 vs. 1.09, p<0.01) compared to healthy subjects. Lower ABI was found in PsA patients than healthy controls, even in those matched with traditional cardiovascular risk factors
Majed Khraishi/2014 [20]	Systematic review	196	Prevalence of cardiovascular risk factors in patients with PsA	-	The prevalence of hypercholesterolemia, obesity, hypertension, diabetes mellitus, anxiety/depression, and coronary heart disease was 61.6, 59.7, 32.7, 13.8, 13.8, and 8.7%, respectively. Overall, the study suggested that PsA, even at early stages, is associated with significant CV comorbidity
Sadhanah Aqashiah Mazlan/2009 [21]	Cross-sectional study	63	To identify the presence of subclinical atherosclerosis in PsA patients attending rheumatology clinics, tertiary hospitals	B sound carotid ultrasound	A significant association between CVS risk and IMT thickness in PsA patients. The positive IMT (IMT >1.00 mm) among PsA was observed in 10 out of 63 patients (15.9%). Otherwise, there was no association in disease activity, disease severity, and DMARDs therapy with IMT thickness in PsA patients

To summarize our results, all nine of our selected studies demonstrated an increased prevalence of subclinical atherosclerosis in PsA patients. The extent of atherosclerosis in PsA patients was assessed using different methods [CIMT, pulse wave velocity (PWV), coronary calcification score (CCS), ankle-brachial index (ABI) values, etc.] in different studies. The most commonly used method was the b-mode ultrasound that measures IMT. CIMT is a useful surrogate and sensitive marker to determine atherosclerosis even in its subclinical stages, and a valid benchmark of IMT was used to determine if it was positive or negatively correlated. As for CVS risk factors, the correlation of IMT with CVS risk factors such as hypertension, diabetes, erythrocyte sedimentation rate (ESR), c-reactive protein (CRP), and hyperlipidemia was found to be significant in all nine studies. These risk factors have been proven to be strong predictors of CVS risk and morbidity and were used in the nine studies selected. 

Bias Evaluation and Data Explication

The quality appraisal was done using the Newcastle-Ottawa scale for the included nine studies. Only moderate-to-high quality studies were included in the final analysis. Table 2 shows the results of the quality appraisal of the included nine studies [1-4,6,14,17,20,21].

**Table 2:** The quality appraisal of studies included in this analysis Selection: (1) case definition adequate?; (2) representativeness of the case; (3) selection of controls; (4) definition of controls (1 point for each question asked for selection) Comparability: 1 point if only cases were studied; 2 points if both cases and controls were studied and compared Outcome: (1) ascertainment of outcome; (2) the same method of ascertainment for controls; (3) non-response rate (1 point for each statement asked regarding outcome)

References	Tool used	Selection				Comparability	Outcome			Total
		1	2	3	4	2 (points)	1	2	3	9
Şule Apraş Bilgen/2018 [1]	Newcastle-Ottawa scale	1	1	1	1	2	1	1		8
Oded Kimhi/2007 [2]	Newcastle-Ottawa scale	1	1	1	1	2	1	1		8
Mehmet Soy/2009 [3]	Newcastle-Ottawa scale	1	1	1	1	2	1	1		8
Yiu KH/2012 [4]	Newcastle-Ottawa scale	1	1	1	1	2	1	1		8
Lihi Eder/2008 [6]	Newcastle-Ottawa scale	1	1	1	1	2	1	1		8
Ivan Gruev/2015 [14]	Newcastle-Ottawa scale	1	1	1	1	2		1		7
Serhad Bilim/2021 [17]	Newcastle-Ottawa scale	1	1	1	1	2	1	1		8
Majed Khraishi/2014 [20]	Newcastle-Ottawa scale	1	1	1	1	2	1	1		8
Sadhanah Aqashiah Mazlan/2009 [21]	Newcastle-Ottawa scale	1	1	1	1	2	1	1		8

Discussion

Psoriasis and Atherosclerosis: Shared Pathomechanisms

Atherosclerosis is an immunoinflammatory disease affecting arteries of different sizes, and various studies show that vascular inflammation has a vital role in the development of atherosclerosis [8]. Disorders linked with inflammatory properties such as SLE and rheumatoid arthritis have recently been shown to be linked to accelerated atherosclerosis. PSO is also a chronic inflammatory disorder; however, very little research has been done on the link between PSO and subclinical atherosclerosis, which is what our study was aimed at [3].

Various different pathophysiologic mechanisms can explain the link between both PSO and subclinical atherosclerosis. Systemic inflammation overall has a huge role in both diseases. PSO is a chronic inflammatory disease of T helper type I cells and shares similar pathophysiology to atherosclerosis and plaque rupture, leading to acute vascular events [4]. Also, studies have shown that patients with PSO have an increased prevalence of CV risk factors versus normal patients [8]. In the development of atherosclerosis, angiogenesis and increased oxidative stress play a big role, and PSO has both these in its development [8].

Eder et al. have emphasized the pathomechanisms of PSO and CVDs. Initial systemic analyses on disease concomitance denoted an association of PSO with CVD, along with other diseases that serve as risk factors for atherosclerosis, such as diabetes mellitus or obesity. The authors observed a conspicuous pattern of associated diseases in patients diagnosed with PSO, hypothesizing a genetically determined selection [9]. In contrast, many studies have shown that PSO's genetic control is independent of atherosclerosis, as in a study done in 2013 by Gupta et al.; the study was done to analyze if PSO, metabolic syndrome, and coronary heart disease shared susceptibility to loci. The study found that the genetic control of PSO is almost completely independent from both metabolic syndrome and coronary heart disease and in contrast, metabolic syndrome and coronary heart disease share 10 common loci [10]. Therefore, the association observed cannot satisfyingly be explained by shared genetics.

However, the epidemiological evidence has been summarized in favor of the association between PSO and subclinical atherosclerosis. This is due to the common inflammatory pathway and the development of lesions as mutual beginning steps in the development of plaque formation in atherosclerosis and PSO, supporting the concept of accelerated atherosclerosis in association with chronic systemic inflammation in diseases such as PsA [11]. These associations, however, do not prove PSO to be an independent risk factor for atherosclerosis, as suggested by the majority of epidemiologic studies [9].

**Figure 2:**
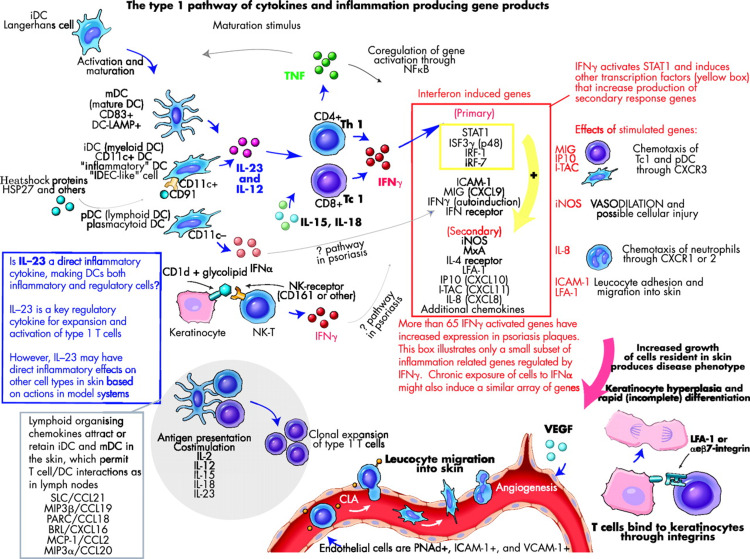
Alternative pathways of leucocyte activation that converge to activate type 1 inflammatory genes which, in turn, regulate end-stage inflammation in the skin and the appearance of the psoriasis phenotype BRL: Bonzo receptor ligand; DC: dendritic cell; ICAM: intercellular adhesion molecule; ITAC: interferon-inducible T cell α chemoattractant; IL: interleukin; IFN: interferon; iNOS, inducible nitric oxide synthase; IP10: interferon-inducible protein 10; IRF: interferon regulatory factor; ISF: interferon-stimulated factor; LFA-1: leucocyte function-associated antigen-1; MCP: monocyte chemoattractant protein; MIG: monokine induced by interferon γ; MIP: macrophage inflammatory protein; MxA: interferon-induced cellular resistance mediator protein; NF: nuclear factor; NK: natural killer (cell); PARC: pulmonary and activation-regulated chemokine; PNAd: peripheral node addressin; TNF: tumor necrosis factor; SLC: secondary lymphoid tissue chemokine Reprinted with permission from Krueger et al. [13]

To prove this and explain that psoriatic inflammation drives atherosclerosis independently from other CV risk factors, the concept of "psoriatic march" was used. According to this concept, PSO is a chronic systemic inflammatory disorder, as evidenced by elevated biomarkers of systemic inflammation. Other than the elevation of classical markers for systemic inflammation, two other mediators - resistin and leptin - are also elevated, and they belong to a family called adipokines. Resistin and leptin are insulin-antagonizing adipokines. Together, the adipokine milieu in patients' blood with PSO is strikingly similar to that of prediabetic individuals and signals a state of insulin resistance [12]. In endothelial cells, insulin resistance is thought to induce endothelial dysfunction and vascular stiffness at the functional level. Several studies have shown evidence for endothelial dysfunction. In particular, flow-mediated vascular dilation was impaired [12]. This mechanism drives atherosclerosis, which ultimately causes CVDs such as MI and stroke. Figure 2 provides an overview of the type I pathway of cytokines and inflammation that occurs in PSO.

Methods Used in Evaluating Endothelial Dysfunction in PsA Patients

Impaired endothelial dysfunction is a precursor of atherosclerosis and has continuously been affiliated with higher CV risk. Different studies have used different methods to assess the endothelial dysfunction in PsA patients. Ultrasonography (b mode) was the most commonly used method we saw in most studies. Among studies using ultrasonography, a study by Apraş Bilgen et al. measured the flow-mediated dilatation (FMD) and CIMT both by ultrasonography, and results showed that PsA and rheumatoid arthritis patients demonstrated significantly lower FMD values compared with healthy controls [1]. CIMT, on the other hand, was only elevated in rheumatoid arthritis patients compared with healthy controls and was similar between PsA patients and healthy controls [1]. However, another study by Yiu et al. showed that patients with PSO had a higher prevalence of subclinical atherosclerosis. CIMT was significantly higher in PSO patients than controls; this was measured again using a high-resolution ultrasound [2]. Greuv et al. did a study in 2015 and used a carotid b mode ultrasound to measure IMT. Results showed that patients with PsA, despite having better CV risk profiles than their controls (hypertensive patients), had higher IMT values; the study also found a significant correlation between IMT and PsA patients [14].

Even though CIMT is a method being used to assess atherosclerosis, evidence of its correlation with future CV events is narrow. A recent study, by Sobchak et al. in 2019, was done to assess if patients with PsA being evaluated for subclinical atherosclerosis by CIMT and total plaque area (TPA) could predict future CV events. The results showed that the rate of developing a first CV event during the study period was 1.11 events per 100 patient-years (95% CI: 0.74-1.67) [15]. The study concluded that combining vascular imaging data with information on traditional CV risk factors could improve CV risk stratification accuracy in patients with PsA and therefore facilitate earlier initiation of appropriate treatment to reduce CV events.

Newer modalities such as using coronary artery calcification (CAC) to assess subclinical atherosclerosis have recently gained popularity. A study was done by Geisel et al. in 2017 to compare the predictive value of CAC, CIMT, and ankle-brachial index (ABI) to assess which of the three markers improved CV risk discrimination best and in which risk group; the study concluded that CAC provides the best discrimination of risk compared with CIMT and ABI, particularly in the intermediate-risk group, whereas CIMT may be an alternative measure for reassurance in the low-risk group [16].

**Figure 3:**
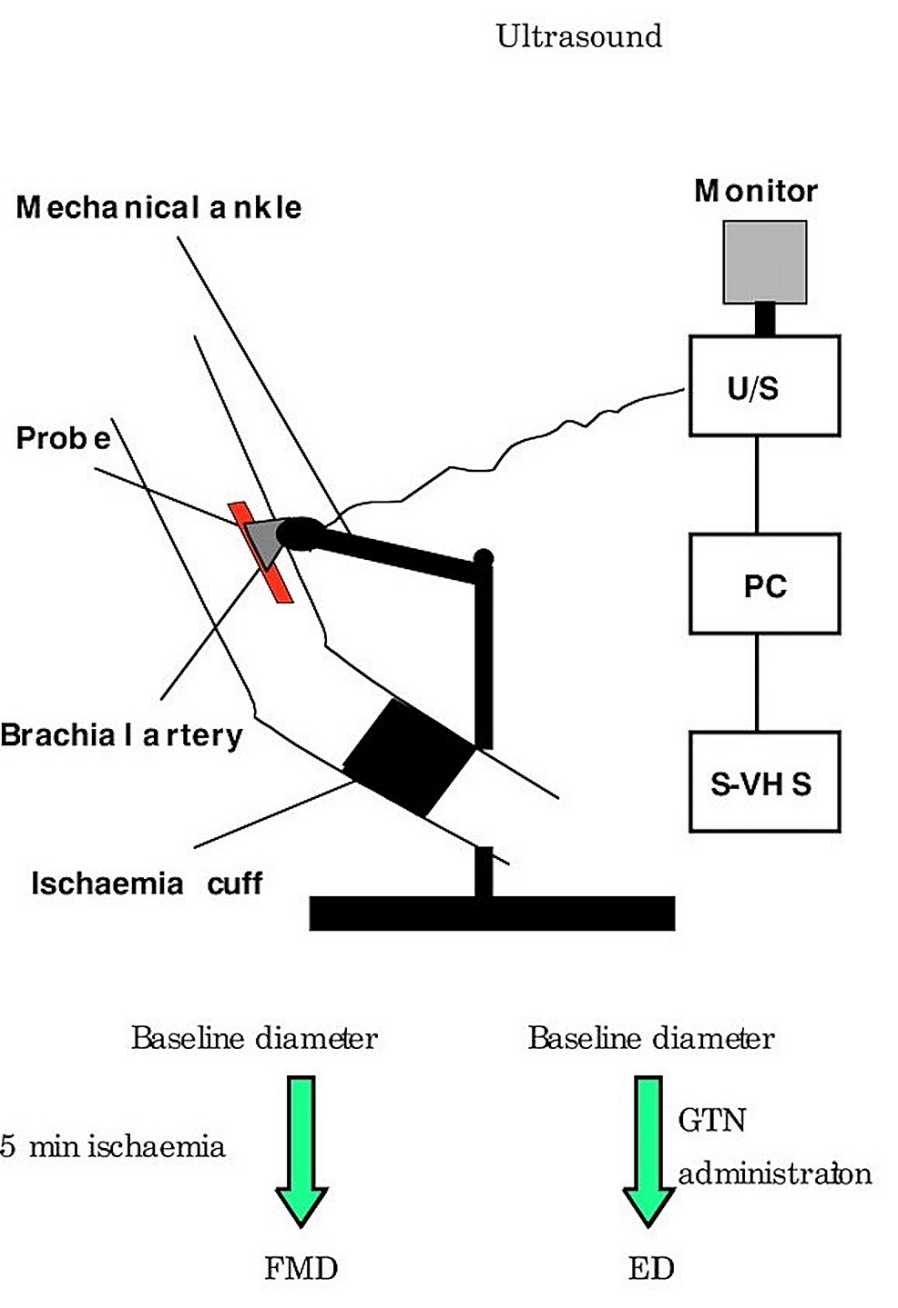
Technical aspects of the non-invasive methods for evaluating endothelial function. Flow-mediated dilation is evaluated by the use of high-resolution ultrasound. FMD: flow-mediated dilation; GTN: glyceryl trinitrate; PC: personal computer; U/S: high-resolution ultrasound Reprinted with permission from Tousoulis et al. [18]

Among other methods, one study by Soy et al. evaluated the prevalence of atherosclerosis in patients with PSO by measuring the carotid-femoral PWV and found that PSO patients had a significantly higher mean PWV than normal patients [3]. Another study by BİLİM et al. used a Doppler probe and a standard blood pressure cuff to measure the ABI values for each patient [17]. They found that patients with PSO had a lower ABI than healthy controls. ABI is a very economical tool and a quite easy method to assess patients for atherosclerosis; however, the study did show that even though it is a sensitive method for detecting atherosclerosis, it can change in daily activities despite resting before measurement [17]. Figure 3 explains the process by which ultrasounds are used in measuring endothelial function.

CV Risk Factors/CV Mortality and PsA

Many previous studies have shown that patients with PsA have a higher prevalence of CVD risk factors than healthy patients. A cross-sectional study by Neimann et al. in 2006 showed that patients with mild and severe PSO had a higher CVS risk profile compared to healthy patients: diabetes mellitus (7.1%, 4.4%, and 3.3%), hypertension (20.0%, 14.7%, and 11.9%), hyperlipidemia (6.0%, 4.7%, and 3.3%), obesity (20.7%, 15.8%and 13.2%), and smoking (30.1%, 28.0%, and 21.3%), respectively [19].

Another study by Yiu et al. in 2012 also found that patients with PSO had a higher body mass index (BMI) than healthy controls. However, they could not find a statistical significance between diabetes, hypertension, smoking, hypercholesterolemia, and PSO due to their small sample size and limited statistical power [4]. This study also found that only patients with PSO having CVS risk factors had a significant correlation between subclinical atherosclerosis and PSO. Those patients with no CVS risk factors had no significant correlation, which suggested that this correlation could be partly due to clustering CVS risk factors.

For PsA patients in particular, although less data is present, studies have shown an increase in CV risk factors and CV morbidity compared to healthy patients or patients with PSO alone. A study was conducted by Khraishi et al. in 2014 showed that the prevalence of hypertension, anxiety, diabetes, and heart disease were all higher in PsA patients [20]. They also found that obesity and hypercholesterolemia were way more prevalent in their study than in other similar studies and attributed this to the regional differences in nutrition. Their study, however, showed no significant correlation between CV mortality in early vs. established PsA patients and suggested that having a longer duration of inflammation is not associated with an increased prevalence of CV mortality [20]. Another study by Eder et al. in 2008 showed that no significant differences in the distribution of diabetes and hypertension were found between the PsA patients and controls [6]. Therefore, it was hypothesized that additional risk factors other than these two, which are probably linked to the immune dysfunction of PsA, may have a role in driving the atherosclerotic process [6].

It has been proposed that a combination of both traditional CV risk factors and disease activity is associated with increased CV risk in PsA patients [21,22]. A recent systematic review and meta-analysis of observational studies showed that PsA patients have a 43% increased risk of CVD and a 55% increased risk of developing incidental CV events compared with the general population and it was also found that the magnitude of the increased risk is similar to that observed in patients with severe PSO [23]. Even though the increased CV risk is being well acknowledged in PsA patients, it is still not being accurately apprehended by traditional risk assessment, and there is a need to develop appropriate risk factor assessments and screening for CVD in this population [24].

The effects of the medication for the treatment of PSO and PsA on CV risk are intricate. While some medications may show favorable effects on CV risk by reducing inflammation, nonsteroidal anti-inflammatory drugs (NSAIDs) and corticosteroids have been associated with elevated cardiac risk among the general population and in rheumatic patients [25,26,27]. The concept of the suppression of inflammation in order to reduce CV risk has been tested in non-rheumatic patients, which showed conflicting results. Two recent RCTs on high-risk patients displayed a reduction in CV risk with IL-1 inhibition (canakinumab) and colchicine contrasted with placebo [28-30]. However, an additional trial with methotrexate displayed negative results [30]. For patients diagnosed with rheumatic diseases like PsA, the suppression of inflammation is the prime treatment objective, and hence it is unethical to conduct similar trials just for a simple analysis of the anti-rheumatic effect.

There is increasing evidence to show that treatment with TNF inhibitors (TNFi) is associated with a diminished risk of developing CVD in patients with PSO and PsA, with a depletion in CV events by approximately 30% among patients using TNFi contrasted against those on non-biologic DMARDs or phototherapy [25,31]. This protective effect may be conciliated by the reduction of vascular inflammation, which may eventually inhibit atherosclerotic plaque progression among PsA patients treated with TNFi [32]. However, long-term real-world observational data are required to precisely quantify CV risk in PsA. Taken together, these data favor the safety and potential cardio-protective effect of TNFi in PSO patients. Further research needs to be conducted to gain information regarding newer classes of biologics.

Limitations

We carried out a systematic review rather than a meta-analysis of the selected studies due to the significant heterogeneity of our topic. This study has some limitations. We did not have full-text access to some of our articles and therefore could not make the best use of them. We could not find any RCTs that fulfilled the selection criteria. In the studies we chose, the sample sizes were relatively small, which could have affected the results and limited their reliability. Finally, we mainly focused on patients with joint manifestations of the disease. Hence, it cannot be ruled out that skin manifestations may have contributed to the high prevalence of atherosclerosis in our group of patients.

## Conclusions

Based on the findings of the reviewed papers, we found that there is an increased risk of subclinical atherosclerosis in PsA patients. These findings are most likely due to the chronic inflammatory status in the PsA disease process, which leads to the increased occurrence of early atherosclerotic changes seen in these patients. Additional research is warranted to determine whether anti-inflammatory treatment may be useful in reducing the risk of atherosclerosis in patients with PsA. CV risk factors that hasten atherosclerosis and CV events are observed more frequently in patients with PsA. More extensive research may be warranted to understand the mechanism behind this association and develop better strategies to improve outcomes related to CV mortality.
